# Causal effects between gut microbiota and coronary heart disease: A Mendelian randomization study

**DOI:** 10.1097/MD.0000000000046282

**Published:** 2025-11-28

**Authors:** Huanyu Chen, Cuicui Zhang, Gengzhen Yao, Xu Zou, Guangming Pan

**Affiliations:** a The Second Clinical Medical College, Guangzhou University of Chinese Medicine, Guangzhou, China; b Department of Cardiology, Guangdong Provincial Hospital of Traditional Chinese Medicine, Guangzhou, China.

**Keywords:** coronary heart disease, genome-wide association study, gut microbiota, inverse-variance weighting, Mendelian randomization

## Abstract

Therapeutic interventions targeting gut microbiota (GM) are promising strategies for the prevention and treatment of coronary heart disease (CHD). Numerous studies have demonstrated a correlation between the GM and CHD. However, these findings do not describe a causal relationship between GM and CHD. Using a bidirectional 2-sample Mendelian Randomization (MR) analysis approach, we utilized a genome-wide association study dataset from the MiBioGen Consortium (N = 18,340) and CHD data from the CARDIoGRAMplusC4D 1000 Genome-based genome-wide association study meta-analysis (60,801 cases and 123,504 controls) to investigate the causal link between GM and CHD. Methods such as inverse-variance weighted, MR-Egger, weighted median, and weighted mode were used to assess causality. Sensitivity analyses, including Cochran Q statistic, MR-Egger intercept, MR-PRESSO global test, and leave-one-out analysis, were used to detect heterogeneity and horizontal pleiotropy. Furthermore, a reverse MR analysis was performed to evaluate the potential for reverse causality. The genus *Butyricicoccus* and order *Victivallales* demonstrated a significant protective effect against CHD at the locus-wide significance level, whereas the genera *Clostridium (innocuum* species), *Oxalobacter*, and *Turicibacter* were associated with an increased risk of CHD. The sensitivity analysis did not yield any substantial indications of pleiotropy or heterogeneity. We describe a simultaneous cause-and-effect relationship between GM and CHD, and expand the range of bacterial taxa that exhibit a causal connection with CHD. These bacterial taxa can serve as innovative biomarkers to facilitate targeted therapeutic interventions for CHD and enhance our understanding of the intricate “gut-heart axis.”

## 1. Introduction

Coronary artery stenosis and inadequate blood supply contribute to myocardial dysfunction, leading to the onset of coronary heart disease (CHD) and organic lesion formation.^[1]^ Despite significant progress in the prevention, diagnosis, and treatment of CHD, the mortality rate remains high, making it an ongoing clinical challenge that cannot be ignored. The high incidence, disability, and mortality associated with CHD have imposed serious economic and health burdens in countries worldwide.^[2–4]^ Hence, prioritizing the development of novel therapy development is crucial for mitigating CHD.

Gut microbiota (GM) encompasses the complete assemblage of bacteria, archaea, viruses, and protozoans that inhabit the gastrointestinal tract. Bacteria represented the most essential phyla, including Bacteroidetes and Firmicutes, followed by Proteobacteria and Actinobacteria, respectively. These 4 phyla accounted for > 90% of the GM.^[5,6]^

As a pivotal active component of the intestinal microecosystem, the intestinal microbiota has a symbiotic relationship with intestinal mucosa. It not only fulfills crucial functions such as food digestion, intestinal safeguarding, and vitamin synthesis, but also influences the pathophysiological processes associated with cardiovascular diseases via microbial metabolites and impacts the endocrine system.^[7,8]^

Environmental and lifestyle factors^[9]^ have a substantial impact on the intestinal microbiota, and coexistence with the host promotes commensal bacterial development, while simultaneously inhibiting opportunistic pathogen propagation.^[10]^ Substantial alterations in the structure and composition of intestinal microbiota have been identified in patients diagnosed with coronary artery disease (CAD).^[11]^

A study conducted by Liu et al observed that the levels of *Weberia*, *Haemophilus*, and *Klebsiella* were higher in individuals with severe CAD than in those without the disease. These bacteria are strongly associated with CAD onset and progression.^[12,13]^ In addition, there was a significant correlation between CHD and intestinal metabolites. Fang et al discovered a notable correlation between an imbalanced GM, the presence of phenylacetylglutamide (PAGIn) derived from the microbiome, and the development of intrastent stenosis in individuals diagnosed with CHD.^[14]^ Compared to healthy individuals, patients with CHD have higher levels of trimethylamine N-oxide (TMAO), which is closely associated with chest pain, coronary atherosclerosis burden, and ACS.^[15,16]^ An increasing number of researchers are currently investigating the association between GM and CHD.^[17]^ However, the present association relies primarily on observational studies with restricted sample sizes and confounding factors, which require further investigation.

Mendelian randomization (MR) uses genetic variants that exhibit strong associations with exposure as instrumental variables (IVs) to infer causal relationships.^[18,19]^ Owing to the use of DNA genotypes for random assignment by prioritizing genotype formation over disease, MR methodology can effectively mitigate potential confounding factors, reverse causality, and other sources of bias that may impact the estimated associations.^[20–22]^ Multiple genome-wide association studies were conducted to explore how host loci influence the prevalence of different gut bacterial types, including combined data for 211 bacterial taxa.^[23]^ This enabled the assessment of potential impacts on various disease outcomes by employing the MR method to examine the causal effects of human genetic modifications. Furthermore, research has focused on genetically modified organisms and CHD. To improve our understanding of the causal association between GM and CHD, we incorporated various taxonomic levels (phylum, class, order, family, and genus) of bacterial taxa and used updated GWAS summary data for CHD with a larger sample size. Additionally, we conducted MR analyses in the reverse direction to rule out the possibility that CHD had a causal effect on the GM.

## 2. Materials and methods

### 2.1. Study design

**Figure 1.**
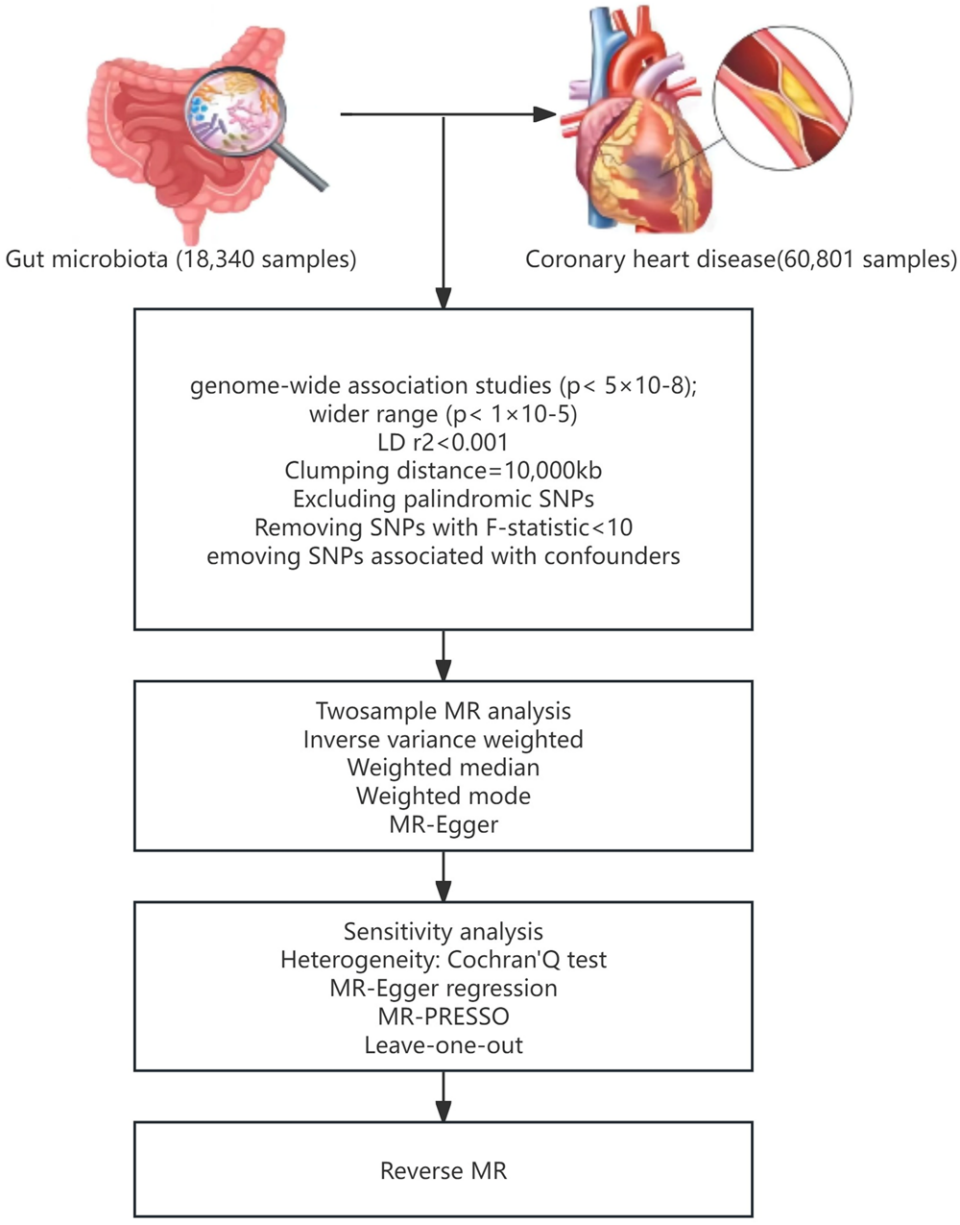
MR analysis investigating the impact of GM on CHD using various methods. CHD = coronary heart disease, GM = gut microbiota, MR = Mendelian randomization.

This study employed a 2-sample MR approach to assess the causal associations between GM and CHD. The overall study procedure is illustrated in Figure 1. To ensure outcome dependability acquired via MR, it is imperative to satisfy 3 fundamental assumptions. First, the IVs used in this analysis demonstrated a significant association with exposure; these IVs were not influenced by potential confounding variables that may impact both the result and level of exposure. Additionally, the impact of these IVs on the outcome occurs solely through their effect on exposure without the involvement of other causal pathways.The second and third were formulated to guarantee the study’s independence, which can be assessed using a battery of statistical methodologies. Finally, we employed reverse MR to explore the potential bidirectional relationship between CHD and the GM.

### 2.2. Data sources

GM-related GWAS data were sourced from the international MiBioGen consortium, which executed a comprehensive, multi-ethnic, genome-wide meta-analysis to explore the link between human autosomal genetic variations and the gut microbiome.^[24,25]^ This meta-analysis included 18,340 participants across 24 cohorts from the United States, Canada, and Europe. After adjustment for sex, age, technical covariates, and genetic principal components, microbiome quantitative trait loci (mbQTL) analysis yielded comprehensive GWAS statistics for 211 microbial groups. These included 9 phyla, 16 classes, 20 orders, 35 families (including 3 unidentified), and 131 genera (12 unidentified). This study cataloged 211 bacterial taxa and identified 122,110 variant loci across 5 taxonomic levels: phylum, class, order, family, and genus. After excluding the 15 genera, the dataset was refined to 196 taxa. The CHD GWAS dataset was derived from the CARDIoGRAMplusC4D 1000 Genome-based meta-analysis, comprising 60,801 cases and 123,504 controls of predominantly European ancestry.^[26]^ This large-scale consortium study aggregated data from 48 studies and is widely used for CAD research because of its robust sample size and comprehensive genotyping strategy.

### 2.3. Instrumental variable selection

To ensure the accuracy of the IVs, we conducted stringent quality control of the selected single nucleotide polymorphisms (SNPs). Given the limited number of SNPs reaching genome-wide significance (*P* < 5 × 10^–8^), we adopted a locus-wide significance threshold *(P* < 1 × 10^–5^), which has been widely applied in previous MR studies to enhance instrument coverage, while maintaining validity.^[26]^ This approach is particularly appropriate when few genome-wide significant variants are available and weak instrument bias is mitigated by excluding SNPs with F-statistics < 10. All included SNPs demonstrated F-statistics > 10, indicating adequate strength and reduced likelihood of weak instrument bias.^[27]^ To prevent linkage disequilibrium related to GM, we performed clumping techniques on the specified SNPs (R^2^ < 0.001, clustering distance = 10000 kb). To ensure the exclusion of palindromic SNPs, measures were implemented to eliminate any potential effects associated with identical alleles and risk factors pertaining to these outcomes using a phenoscanner to investigate and filter out any SNPs linked to potential confounding variables or factors such as high blood pressure, diabetes, and obesity. To address the potential bias arising from weak IVs, we calculated the F-statistic for each bacterial taxon, using the following formula:


$$\begin{array}{l} F^{{}^{1}/{}_{4}}=\frac{R^{2}\times \left(n-k-1\right)}{k\times \delta 1-R^{2}} \end{array}$$

The value of R^2^ signifies the extent to which the selected SNPs account for variability in exposure, with *n* representing the sample size and number of IVs included.^[28]^ IVs exhibiting *F*-statistics <10 were deemed inadequate and thus omitted from the analysis. To ascertain the influence of CHD on GM composition, we employed genetic variants linked to CHD at genome-wide significance (*P* < 5 × 10^–8^) as instruments for reverse MR analysis, adhering to the same filtering criteria outlined previously.

### 2.4. MR analysis

The inverse-variance weighting (IVW) method was used to infer the potential causal link between GM and CHD, while accounting for potential horizontal pleiotropy. To strengthen the reliability of our findings, we used supplementary approaches, such as the MR-Egger and weighted median (WM) methods with IVW. These were selected based on their capacity to satisfy the underlying assumptions of their corresponding models. Specifically, for the WM method, a minimum of 50% of SNPs exhibit no pleiotropic effects,^[29]^ and if the occurrence of pleiotropy in MR-Egger inferences is ≤ 50%, SNP findings remain reliable.^[30]^ In terms of the significance of our findings, we considered the causal probability < 0.05 between exposure and outcome to be nominally significant if supported by at least 1 additional method.

### 2.5. Sensitivity analysis

Given the diversity of subjects and methods used in our study, heterogeneity in MR analysis was a potential concern. Cochran Q test was used to assess variability among the IVs. A *P*-value >.05 suggested that heterogeneity did not significantly affect the causal estimates, negating the need for further adjustments. Conversely, significant heterogeneity (*P* <.05) warrants the use of the IVW random effects model to address its influence. Additionally, we employed logistic regression to explore potential horizontal pleiotropy among the selected SNPs, accounting for possible confounders and unidentified causal pathways affecting genetic diversity. Instances of significant pleiotropy (*P* <.05) were excluded from the analysis.^[31]^ Given the limitations of MR-Egger regression, including concerns about precision and validity, we utilized MR Pleiotropy RESidual Sum and Outlier (MR-PRESSO) analysis to detect outliers, suggesting pleiotropic bias.^[32]^ This reanalysis confirmed the stability of the findings. To further validate the robustness of our causal inferences, leave-one-out sensitivity analysis was conducted to examine the influence of individual SNPs on the overall results.^[33]^ This involved assessing shifts in the overall estimate upon sequential exclusion of each SNP, with significant deviations indicating potential outliers. These outliers were identified based on predetermined criteria (distance threshold of 0.5 in the 1-out analysis), ensuring the consistency and reliability of our MR findings.

### 2.6. Statistical analysis

Statistical analyses were performed using the R program’s “TwoSampleMR” package (version 4.2.2). To perform the MR-PRESSO test, we utilized the “MR-PRESSO” package. FigDraw (www.figdraw.com) was used to generate data.

### 2.7. Ethics

The aggregate-level data used in this study included publicly available de-identified data approved by ethical standards committees; hence, it was not necessary to obtain further ethical approval.

## 3. Results

### 3.1. IVs associated with GM

Seven datasets were acquired for the MR analysis, detailing coalitions, sample sizes, and populations (Table S1, Supplemental Digital Content, https://links.lww.com/MD/Q798). Significant nucleotide polymorphisms were extracted from GWAS across 9 phyla, 16 classes, 20 orders, 35 families, and 131 genera, using a significance threshold of 1 × 10^–5^. Aggregation and coordination subsequently facilitated MR analysis to investigate causality between each exposure-outcome pair, and instrumental variable analysis confirmed the robustness of our findings, with F-statistics over 10 for all variables, indicating minimal bias from weak instruments (Table S1, Supplemental Digital Content, https://links.lww.com/MD/Q798).

### 3.2. Causality and sensitivity analysis of GM in CHD

**Figure 2.**
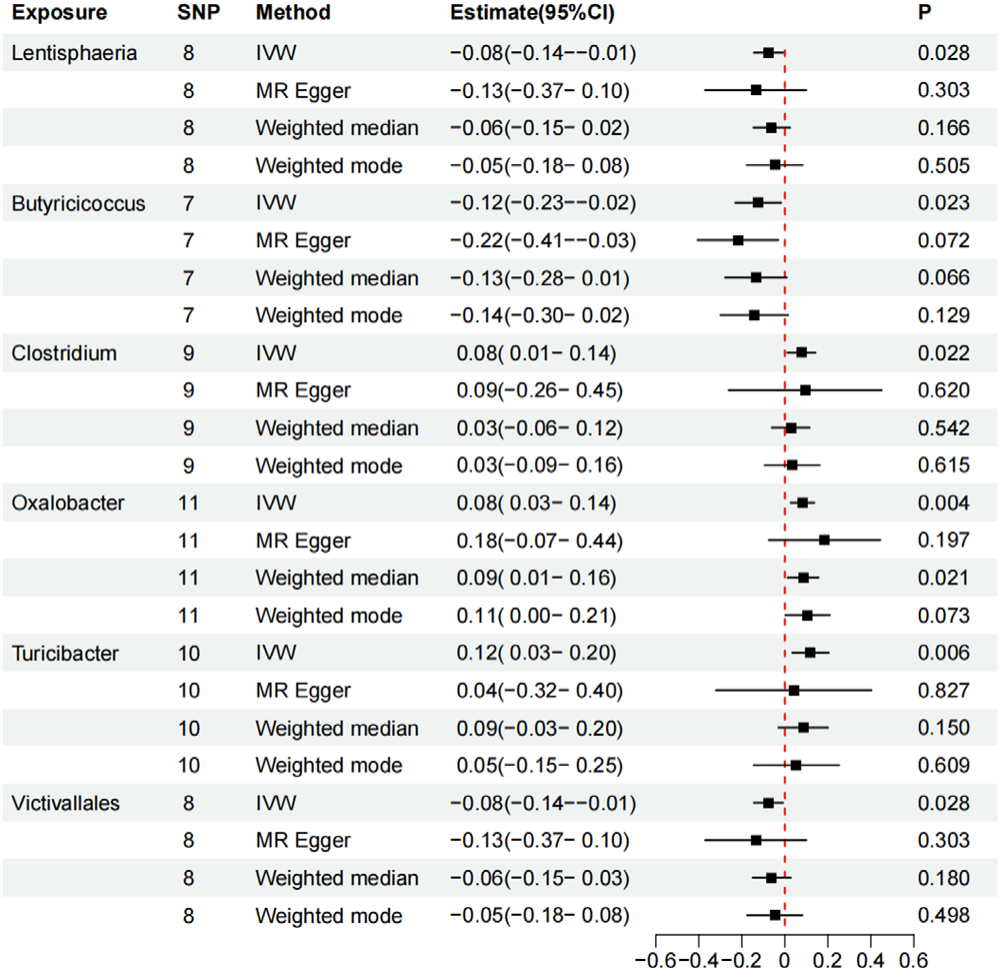
Causal relationship between coronary heart disease risk and gut microbiome: insights from a 2-sample MR study. IVW = inverse-variance weighted method, MR = Mendelian randomization.

**Figure 3.**
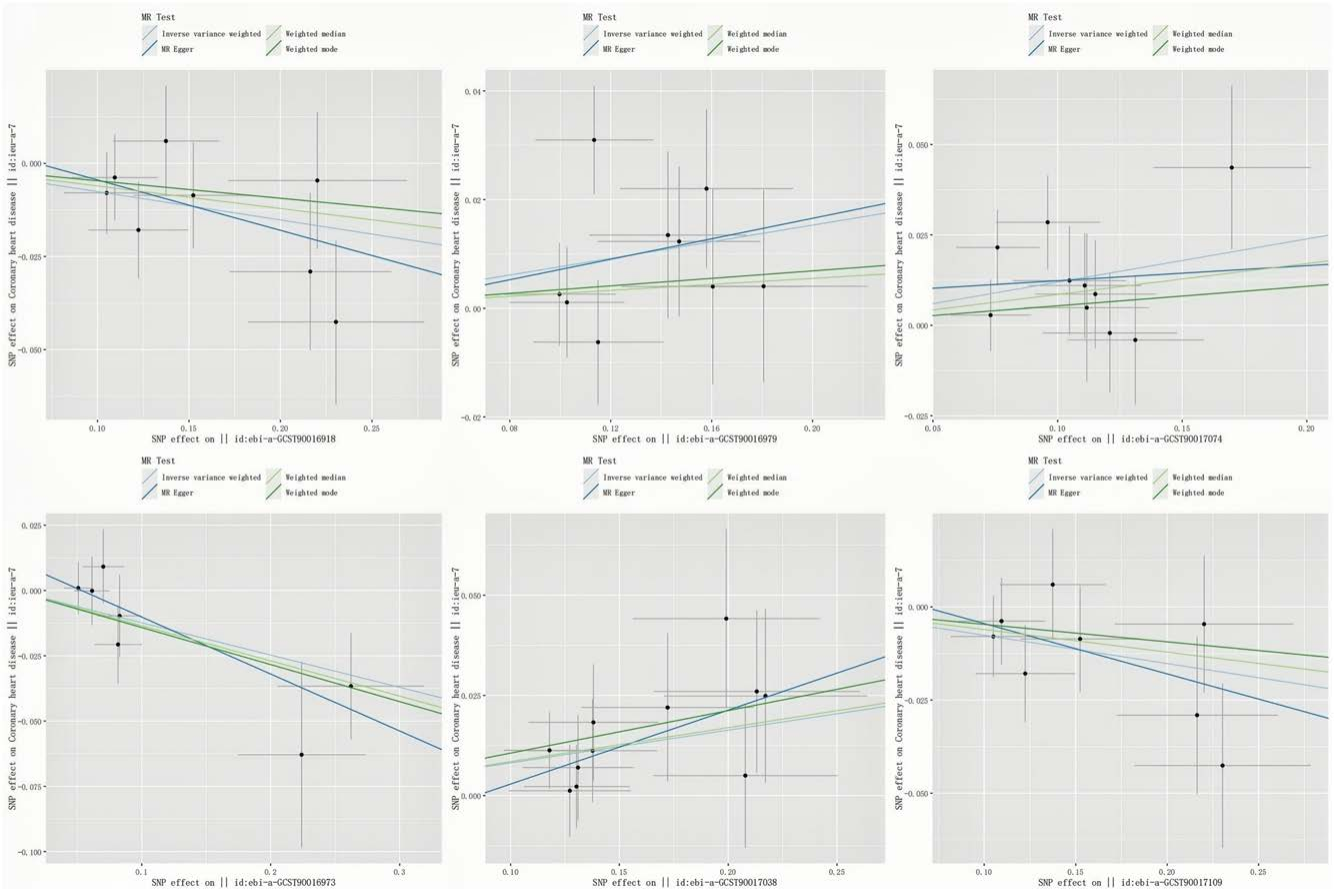
Scatter plot demonstrating the causal influence of GM on coronary heart disease. GM = gut microbiota.

**Figure 4.**
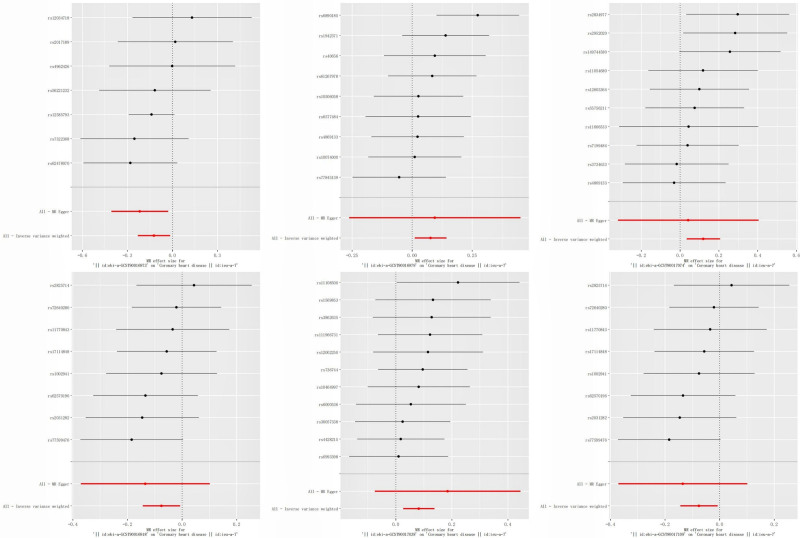
Forest plot depicting the causal link between GM and coronary heart disease. GM = gut microbiota.

Following data preprocessing, MR analyses were conducted for each exposure-outcome pair (GM and CHD) using 4 MR methods: IVW, WM, weighted mode, and MR Egger. The application of MR in principal analysis yielded findings that support an association between 6 specific bacterial taxa and susceptibility to CHD. All IVs met the criteria for solid instruments, and additional sensitivity analyses confirmed the statistical significance of the findings related to these 6 bacterial taxa. Table S2, Supplemental Digital Content, https://links.lww.com/MD/Q798 provides detailed information on single SNPs. Specifically, a greater prevalence of class *Lentisphaeria*, genus *Butyricicoccus*, and order Victivallales demonstrated a protective effect (OR: 0.927, 95% CI: 0.866–0.875, *P* = .028), for class *Lentisphaeria* (OR: 0.884, 95% CI: 0.794–0.983, *P* = .021), for genus *Butyricicoccus* (OR: 0.927, 95% CI: 0.866–0.992, *P* = .028), for order Victivallales, while *Clostridium innocuum*, genus *Oxalobacter*, and genus *Turicibacter* was risk factors for CHD (OR: 1.08, 95% CI: 1.011–1.153, *P* = .022), for genus *Oxalobacter* (OR: 1.085, 95% CI: 1.027–1.147, *P* = .004), for *Clostridium innocuum* (OR: 1.127, 95% CI: 1.035–1.227, *P* = .006, for genus *Turicibacter*) according to the data presented in Table 1 and Figure 2. Table S3, Supplemental Digital Content, https://links.lww.com/MD/Q798 presents the results of all MR studies and sensitivity analyses, and the causal relationship between gut flora and CHD risk, as analyzed using the 4 aforementioned MR methods, is depicted through scatter and forest plots (Figs. 3 and 4). Sensitivity analyses, as presented in Tables S4 and 5, Supplemental Digital Content, https://links.lww.com/MD/Q798, revealed no indications of horizontal pleiotropy according to MR-Egger regression intercepts (*P* > .05). The MR-Egger regression results were further validated using MR-PRESSO, which identified no outliers (Table S6, Supplemental Digital Content, https://links.lww.com/MD/Q798). Leave-one-out analysis (Fig. S1, Supplemental Digital Content, https://links.lww.com/MD/Q799) revealed no influential single SNPs, further supporting the robustness of the findings. Alternative statistical approaches, such as the MR-PRESSO global test and MR-Egger intercept test, did not show any irregularities. These results support the conclusion that outliers do not significantly affect the findings. Overall, the sensitivity analyses conducted in this study suggest that MR analysis is reliable.

**Table 1:** MR analyses investigating the impact of gut microbiota on coronary heart disease using various methodological approaches.

Taxa	GM (exposure)	Trait (outcome)	Nsnp	Methods	Beta	SE	OR (95% CI)	*P*-value	Heterogeneity		Horizontal pleiotropy		
									Cochran Q	*P*-value	Egger intercept	SE	*P*-value
class	Lentisphaeria	CHD	8	Inverse-variance weighted	–0.08	0.03	0.93 (0.87–0.99)	.028	3.979	0.782	0.009	0.018	0.625
				MR Egger	–0.14	0.12	0.87 (0.69–1.11)	.303					
				Weighted median	–0.06	0.04	0.94 (0.86–1.03)	.166					
				Weighted mode	–0.05	0.07	0.95 (0.84–1.09)	.505					
genus	***Butyricicoccus***	CHD	7	Inverse-variance weighted	–0.12	0.05	0.88 (0.79–0.98)	.023	3.964	0.682	0.012	0.010	0.286
				MR Egger	–0.22	0.10	0.80 (0.67–0.97)	.072					
				Weighted median	–0.13	0.07	0.87 (0.76–1.01)	.066					
				Weighted mode	–0.14	0.08	0.87 (0.74–1.02)	.129					
genus	Clostridium innocuum group	CHD	9	Inverse-variance weighted	0.08	0.03	1.08 (1.01–1.15)	.022	8.562	0.381	–0.002	0.023	0.924
				MR Egger	0.09	0.18	1.10 (0.77–1.57)	.620					
				Weighted median	0.03	0.05	1.03 (0.94–1.12)	.542					
				Weighted mode	0.03	0.07	1.04 (0.91–1.18)	.615					
genus	*Oxalobacter*	CHD	11	Inverse-variance weighted	0.08	0.03	1.09 (1.03–1.15)	.004	4.155	0.940	–0.016	0.020	0.447
				MR Egger	0.18	0.13	1.20 (0.93–1.56)	.197					
				Weighted median	0.08	0.04	1.09 (1.01–1.17)	.021					
				Weighted mode	0.11	0.05	1.11 (1.00–1.23)	.073					
genus	Turicibacter	CHD	10	Inverse-variance weighted	0.12	0.04	1.13 (1.03–1.23)	.006	7.201	0.616	0.008	0.019	0.676
				MR Egger	0.04	0.18	1.04 (0.73–1.50)	.827					
				Weighted median	0.09	0.06	1.09 (0.97–1.22)	.150					
				Weighted mode	0.05	0.10	1.06 (0.86–1.29)	.609					
order	Victivallales	CHD	8	Inverse-variance weighted	–0.08	0.03	0.93 (0.87–0.99)	.028	3.979	0.782	0.009	0.018	0.625
				MR Egger	–0.14	0.12	0.87 (0.69–1.11)	.303					
				Weighted median	–0.06	0.05	0.94 (0.86–1.03)	.180					
				Weighted mode	–0.05	0.07	0.95 (0.84–1.09)	.498					

CHD = coronary heart disease, CI = confidence interval, GM = gut microbiota, MR = Mendelian randomization, OR = odds ratio, SE = standard error, SNP = single nucleotide polymorphism.

### 3.3. Reverse MR analysis

To mitigate the potential impact of reverse causality on these findings, reverse MR analysis was conducted to investigate the causal influence of CHD on the GM, thereby confirming the direction of causality. The results are presented in Tables S7 and S8, Supplemental Digital Content, https://links.lww.com/MD/Q798. Notably, of the 6 taxa previously identified in the MR analysis of CHD-related gut flora that were significantly associated with CHD risk, only *Butyricicoccus* exhibited a negative correlation with CHD (OR, 0.949; 95% CI, 0.913–0.986; *P* = .007). The omission sensitivity analysis, as shown in Figure S2, Supplemental Digital Content, https://links.lww.com/MD/Q799, validates the reliability of these reverse MR findings. Furthermore, analyses, including the MR-Egger regression intercept, Cochran Q test (detailed in Tables S9 and 10, Supplemental Digital Content, https://links.lww.com/MD/Q798 and Figures S3 and 4), and MR-PRESSO (Table S11, Supplemental Digital Content, https://links.lww.com/MD/Q798), revealed no significant evidence of horizontal pleiotropy.

## 4. Discussion

We present a groundbreaking effort to use the bidirectional MR method to explore the potential causal link between CHD and GM instead of solely focusing on evaluating the reliability of the analysis through different sensitivity analyses and eliminating interference from reverse causality. Our results suggested that some intestinal bacteria can promote or prevent CHD and its associated risk factors.

Previous studies have confirmed that the association between intestinal microbiota and the occurrence or development of a potential link between GM and CHD remains challenging owing to the inherent constraints on experimental research involving humans. Therefore, our study aimed to evaluate the causal association between CHD and GM by using a bidirectional 2-sample MR approach. In this study, we identified 6 specific microbial taxa. The results of the MR-BA analysis revealed that a high abundance of the class Lentisphaeria, genus *Butyricococcus*, and order Victivallales was a protective factor for CHD, whereas the presence of *Clostridium innocuum*, *Oxalobacter*, and *Turicibacter* was correlated with an elevated susceptibility to CHD. The results of this study show that GM is not only a potential indicator for the early identification of individuals at high risk of CHD but also a breakthrough point for achieving optimized prevention and treatment strategies.

Several observational studies have investigated the relationship between coronary atherosclerosis and GM composition by using metagene sequencing. However, these studies have not been conducted in depth to identify specific microbial species, and the results of differential analysis of GM associated with CHD lack consistency in different studies. For example, Karlsson et al reported an increase in the prevalence of *Collinsella* among individuals diagnosed with CHD. However, the abundances of *Eubacterium* and *Roseburia* decreased.^[34]^ Another comparative study conducted by Wang et al showed that *Eubacteria* and *Roseburia* were significantly enriched in patients with CHD.^[35]^ This lack of homogeneity may be because each part of the gastrointestinal tract has a special microbiota composition,^[36]^ making it more difficult to identify specific pathogenic and nonpathogenic microbiota. This may also be related to insufficient statistical power and insufficient control of confounding variables such as age, ethnic differences in the recruited population, diet, and drugs.^[37]^ The MR study design provides a more reliable analysis by minimizing the influence of confounding variables, which can compensate for these shortcomings. Among the microbial populations with potential causal relationships with CHD identified in this study, *Oxalobacter* was the most significant (OR, 1.085; 95% CI, 1.027–1.147; *P* = .004), and several studies have reported a significant increase in the abundance of oxalate in patients with hypertensive nephropathy. However, the mechanism by which it induces hypertensive nephropathy remains unclear, and may be associated with its involvement in bile acid secretion.^[38,39]^ The model species *Oxalobacter formigenes* has emerged as a prominent subject of kidney stone research in recent years.^[40]^ It is commonly acknowledged that the presence of oxalate hinders the absorption of mucosal and triggers the release of endogenous oxalate in the intestinal mucosa by degrading luminal oxalate.^[41]^ However, the specific mechanism of action of oxalate in CHD remains unclear. Existing data indicate that *Oxalobacter* may increase the risk of CHD.^[42]^ Zheng et al revealed a notable increase in various types of intestinal microbiota such as *Oxalobacter* in individuals diagnosed with CHD.^[43]^ The above research is consistent with our results; however, the researchers did not study or discuss this outcome further. Therefore, it is vital to investigate the causal effects of this species on CHD.

Among the microbiota with a protective effect on CHD investigated in this study, *Butyricoccus* had the highest significance, but its exact mechanism of action on CHD remains unclear and requires further research and verification. Probiotics can effectively improve the symptoms of CHD by enhancing the microbial makeup of the host’s gut, such as by increasing the abundance of *Butyricoccus* and improving the microbial metabolic potential and serum metabolic profile.^[44,45]^ In vitro and animal studies conducted by Huang et al found that *Butyricoccus* has a protective effect against heart failure with a preserved ejection fraction.^[43]^ This mechanism is related to the ability of *Butyricoccus* to downregulate the level of substance-density pro-inflammatory mediators and reduce human inflammation.^[46–50]^ The decreased abundance of butyrate-producing intestinal microorganisms is also a feature of CHD.^[51]^ Therefore, additional GM metabolites must be considered to investigate the potential causal association between CHD and GM. Interestingly, the reverse MR analysis indicated that CHD might exert a protective effect on the presence or abundance of *Butyricoccus*. This consistency across analyses is notable, given that reverse MR is typically employed to investigate the possibility of reverse causation, thereby challenging the notion of reverse causation itself. Observing similar outcomes from both analytical directions may suggest a robust and potentially bidirectional relationship, warranting careful interpretation. It is crucial to acknowledge that the genetic instruments utilized in MR studies should be closely linked to exposure, without being directly associated with the outcome of causal analysis. The reliability of MR findings largely depends on the chosen genetic variants as IVs, which ought to be strongly linked to exposure (levels of *Butyricoccus*) and not to potential confounders or the outcome (CHD), except for the exposure pathway. Moreover, MR studies must consider population stratification, which can obscure the relationship between genetic variation and outcomes. Overall, the observed link between *Butyricoccus* and decreased risk of CHD is intriguing and merits further exploration. Future research should focus on validating these results in diverse populations and elucidating the mechanisms by which *Butyricoccus* may influence cardiovascular health.

Our study had some limitations. Initially, despite the fact that the CHD-associated laws considered in this investigation possess an extensive and well-documented sample size, the data were from 18,340 European minority participants in 24 cohorts from 11 countries; therefore, it is difficult to apply the findings to other ethnic groups. Second, it is not entirely impossible that there may be some potential correlations between the genetic makeup of an individual’s diet and the environment, which could potentially influence our results. Third, a significant proportion of the studies included involved the inclusion of both case and control groups; however, it is crucial to acknowledge that not all participants included in the study underwent stringent quality control measures, and that there may also be variations in the composition of GM and metabolites. Therefore, we must recognize the substantial impact of age on the stability of our findings. Fourth, unobserved pleiotropy cannot be addressed, as in other Mendelian studies. It is crucial to acknowledge that when specific instrumental SNPs demonstrate horizontal pleiotropy, it is imperative to identify and address this phenomenon. For example, when genetic determinants are associated with CHD, the IVW effect estimates may be biased.

Despite these limitations, the present study had several advantages. The extensive dataset used in this study facilitates comprehensive analysis of CHD events, allowing the development of a genetic tool for MR analysis through a robust GWAS. Furthermore, consistent causal estimates obtained from the 6 methods (WM, MR-Egger, MR-PRESSO, Cochrane Q test, and IVW) validated the robustness and reliability of our findings.

## Acknowledgments

The authors extend their sincere appreciation to the members of the MiBioGen Consortium acknowledged in this article, as well as to the investigators of genome-wide association studies, including the CARDIoGRAMplusC4D Consortium, for providing publicly available CHD GWAS summary statistics. We also acknowledge other contributing institutions for their efforts to generate and share the data resources that made this study possible.

## Author contributions

**Conceptualization:** Huanyu Chen.

**Data curation:** Huanyu Chen.

**Formal analysis:** Huanyu Chen.

**Funding acquisition:** Xu Zou, Guangming Pan.

**Investigation:** Gengzhen Yao.

**Methodology:** Huanyu Chen, Gengzhen Yao, Xu Zou, Guangming Pan.

**Visualization:** Huanyu Chen, Cuicui Zhang.

**Writing – original draft:** Huanyu Chen.

**Writing – review & editing:** Xu Zou, Guangming Pan.

## Supplementary Material




