# Supplementary material for: Causal effects between gut microbiota and coronary heart disease: A Mendelian randomization study
Source: Medicine (Baltimore). 2025 Nov 28;104(48):e46282. doi: 10.1097/MD.0000000000046282 (PMC12662416; doi:10.1097/MD.0000000000046282)

Supplementary Figure 1. Leave-one-out analysis results for six bacteria showing statistical significance in MR studies on the relationship between gut microbiota and hypertension.

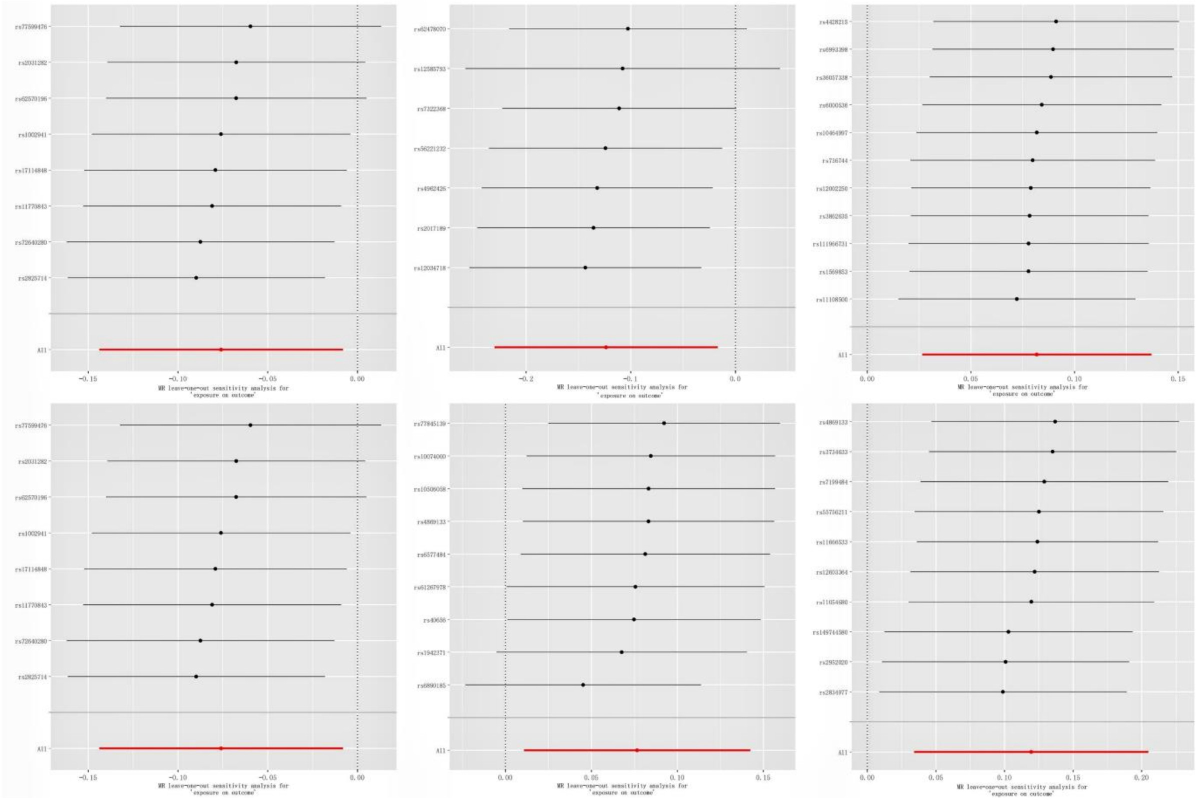

Supplementary Figure 2. Scatter plots illustrating the causal relationship between coronary heart disease and gut microbiota.

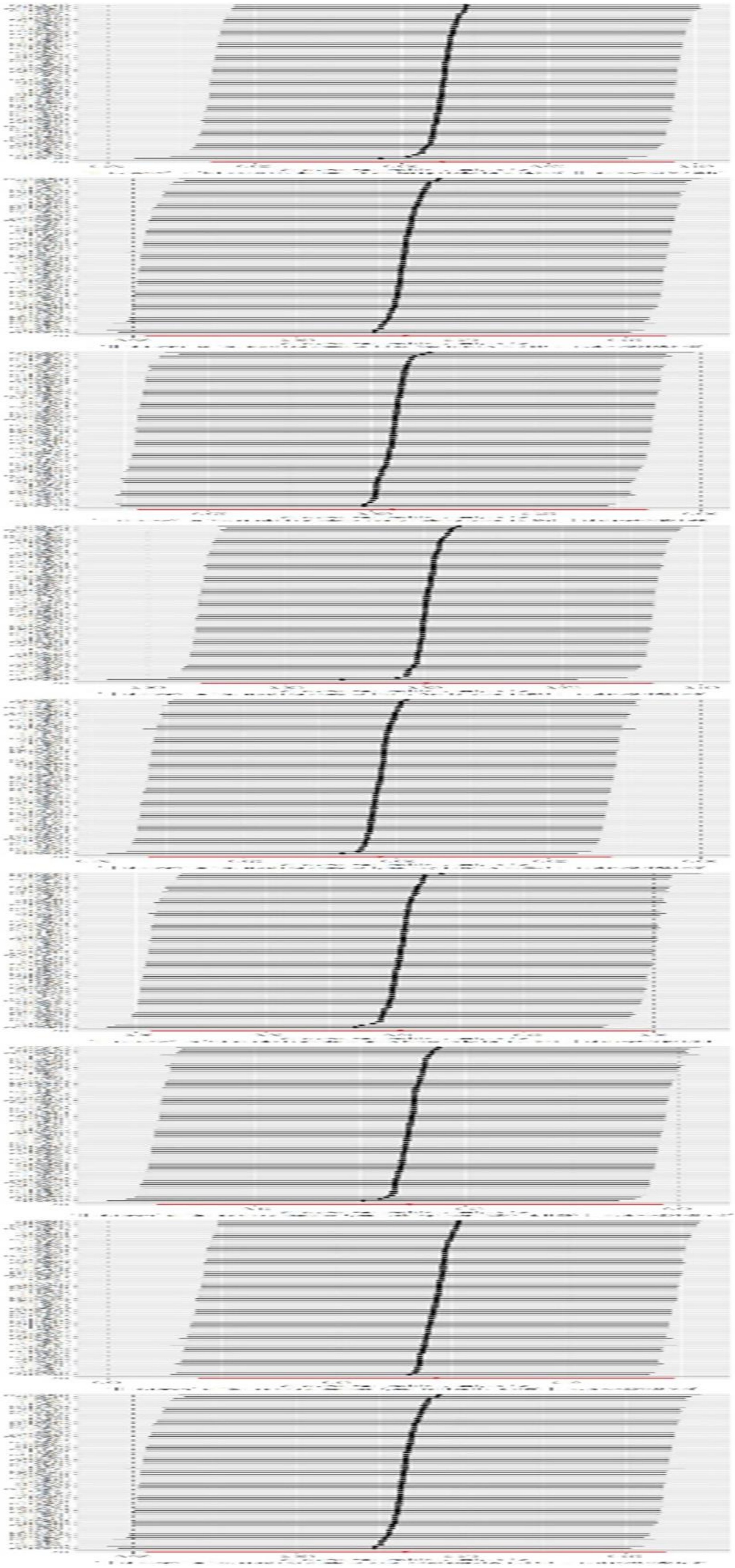

Supplementary Figure 3. Forest plots illustrating the causal relationship between coronary heart disease and gut microbiota.

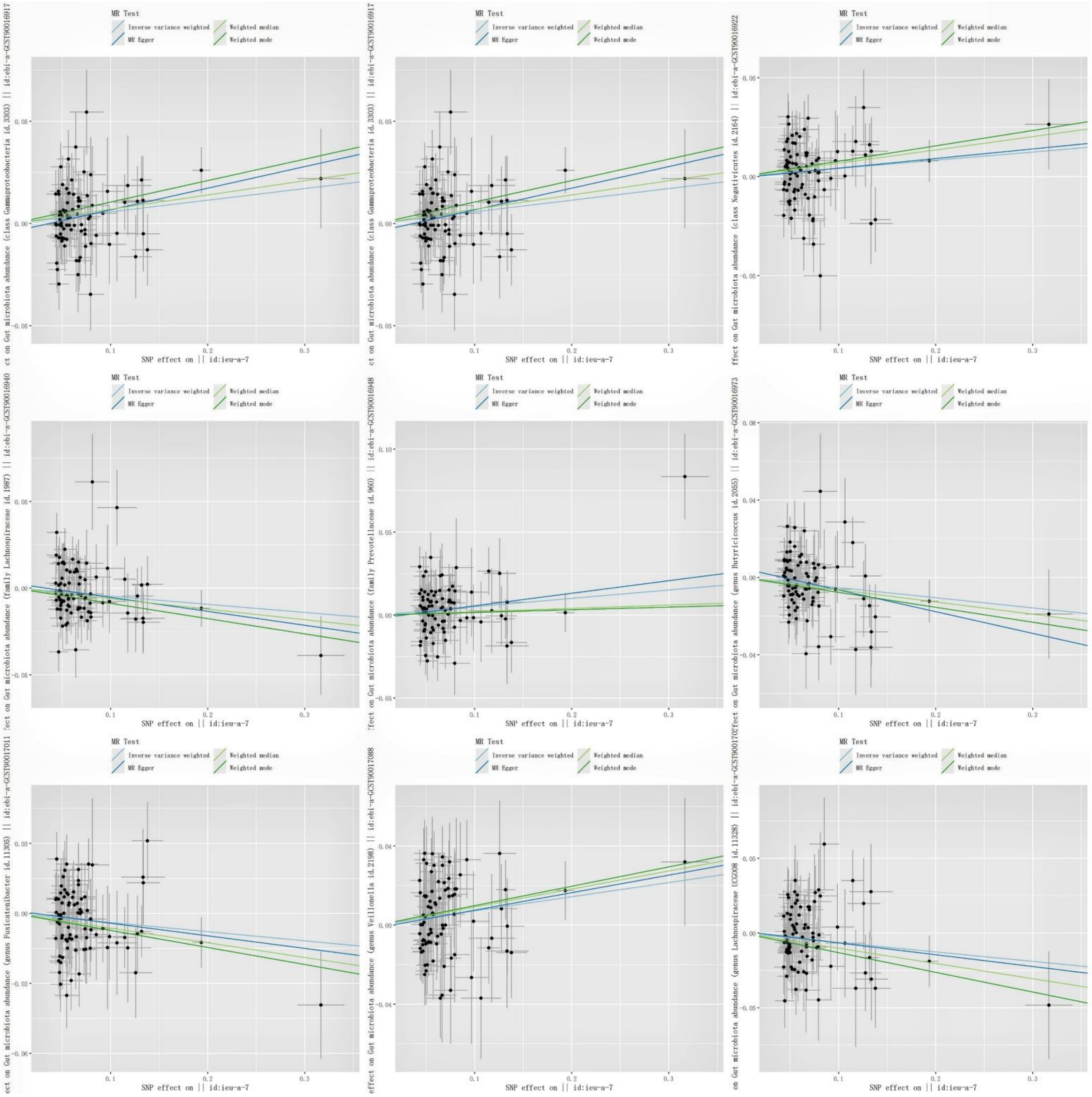

Supplementary Figure 4. Statistically significant outcomes from the leave-one-out analysis in the reverse MR study.

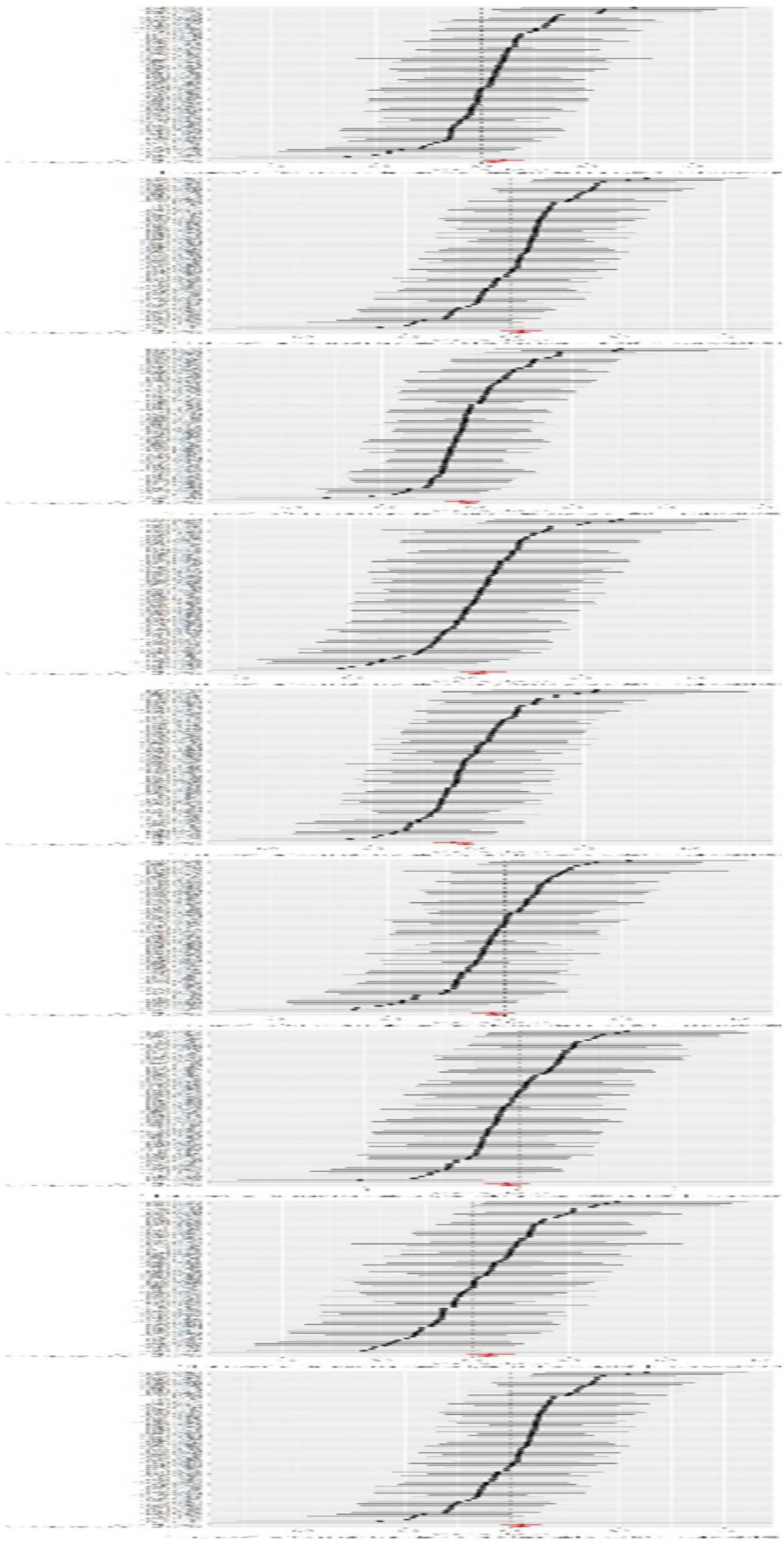

Supplement: Supplementary file 2 [file medi-104-e46282-s002.pdf]
